# Factors relating to the mental health of women who were pregnant at the time of the Great East Japan earthquake: analysis from month 10 to month 48 after the earthquake

**DOI:** 10.1186/s13030-016-0072-6

**Published:** 2016-06-27

**Authors:** Kineko Sato, Maki Oikawa, Mai Hiwatashi, Mari Sato, Nobuko Oyamada

**Affiliations:** Maternal Nursing, Tohoku University Graduate School of Medicine, 2-1 Seiryo-machi, Aoba-ku, Sendai-shi, #981-8575 Japan

**Keywords:** The Great East Japan earthquake, Post-partum depression, GHQ-28

## Abstract

**Background:**

The Great East Japan Earthquake occurred at 2:46 p.m. on March 11, 2011. The epicenter was off the coast of Miyagi prefecture, and the magnitude of the earthquake was 9.0 with a maximum seismic intensity of 7.0. Although it has already been four years, victims continue to have complex problems. In the stricken areas of Miyagi prefecture, almost ten percent of the residents continue to live in temporary housing. Life altering events that force relocation and a change of living environment are known to adversely affect mental health. The purpose of this study was to examine the mental health of mothers of infants who experienced this disaster in Miyagi prefecture.

**Methods:**

We conducted a survey using The Edinburgh Postnatal Depression Scale (10 months) and The General Health Questionnaire-28, an efficient screening tool for psychiatric distress. Eight hundred eighty-six mothers of children born from February to October, 2011 in Miyagi prefecture were surveyed 10, 16, 24, 36 and 48 months after the disaster. Data were analyzed with the use of SPSS 21.0 J for Windows. The study was approval by the review board of ethics at Tohoku University.

**Results:**

The questionnaire was answered by the following number of mothers at the specified months after the disaster: 677 at 10 months, 384 at 16 months, 351 at 24 months, 250 at 36 months and 193 at 48 months. Results at all time periods indicated a high prevalence of psychiatric distress among the mothers surveyed. The percentage of Japanese adults with high-risk GHQ-28 scores is 14 %, thus psychological distress among the subjects in the present study is considerably more widespread. General Health Questionnaire-28 scores were significantly higher for those mothers experiencing dissatisfaction in their marital relationships. We found that mothers have experienced severe mental distress since the disaster, which we think is a possible cause of depression that is leading to poor mental health.

**Conclusion:**

The results indicate that the upheaval caused by the tsunami affected the mental health of the mothers. Psychological distress continued to be prevalent up to four years after the disaster. Different factors were found to be associated with their distress. The most common issues were economic problems, dissatisfaction in the marital relationship, and no support with childcare.

## Background

The Great East Japan Earthquake occurred at 2:46 p.m. on March 11, 2011. The epicenter was off the coast of Miyagi prefecture, and the magnitude of the earthquake was 9.0 with a maximum seismic intensity of 7.0. The giant tsunami that accompanied the earthquake destroyed many houses (around 400,000 houses either fully or partially destroyed) and took many lives (at least 15,000 deaths), causing devastation over a large area of the eastern coast of the Tohoku region of Japan. Although it has been four years after the disaster, 23,132 people in Miyagi prefecture still reside in temporary accommodations (http://www.fdma.go.jp/bn/higaihou_past_jishin.html). In addition, the Fukushima nuclear power plant incident occurred and numerous residents are still unable to return to their hometown. It is difficult to predict when these people will be able to return. Even in these harsh circumstances, 19,126 babies were born in Miyagi prefecture in 2011, and mothers are currently rearing their children in an earthquake-stricken living environment.

Even in times without earthquakes, pregnant women and women who have recently given birth are psychologically susceptible to experiencing a depressed state; in particular, post-partum depression is known to be a problem that may prevent a mother from developing an attachment to her child [1–6]. It is also reported that the post-partum depression of mothers is correlated with and influences the depression of fathers [7]. A study on the effects of the earthquake on the psychophysical condition of mothers and their children was carried out three years after the great Hanshin-Awaji Earthquake site. The study results showed that psychophysical effects of the disaster on mothers and their children from areas with comparatively lesser damage were seen up to more than a year after the earthquake [8]. In addition, significant predictors of postpartum depression associated with the Noto Peninsula earthquake were ‘anxiety towards the earthquake’ and the frequency of childbirth [9].

Accordingly, we recently conducted a continuing survey of women who were pregnant at the time of the Great East Japan Earthquake and those in the first month after childbirth. We surveyed the mothers at 10, 16, 24, 36 and 48 months after the earthquake. Our objective was to assess the physical and mental state of child-rearing mothers after the earthquake to determine what influence it had on their physical and mental health.

## Methods

1. Subjects
  A survey questionnaire was mailed to 3539 women who were either pregnant or in the early postnatal stage (within one month of childbirth) living in Miyagi prefecture at the time of the Great East Japan Earthquake. The study received approval from the Ethical Review Board of Tohoku University. Subjects participated in the survey of their own free will. We informed the subjects in writing that they would not suffer any disadvantage if they did not participate. Subjects who completed the survey questionnaire returned it by mail, with 886 women providing consent to participate (25 % response rate). Although the study began with these 886 women, a gradual decrease in respondents due to large scale relocation was observed, and we had difficulty in contacting them because the mailing addresses for the survey slips changed dramatically over time. However, the subjects who reported were from among these 886 women.
2. Period of survey
  Surveys were conducted in January 2012, July 2012, March 2013, March 2014, and March 2015.
3. Content of survey
  The survey inquired about the attributes and living circumstances of the subjects and their physical and mental state as mothers. To assess their physical and mental condition we used the Edinburgh Postnatal Depression Scale (EPDS) at month 10 after the earthquake, which is useful for the assessing postnatal depressive state. The maximum score on the EPDS is 30 points, and the cutoff score is 9 points in Japan. Respondents scoring 9 or higher are at high risk for postnatal depression and require careful observation. The EPDS can be used for up to six months after giving birth as a measure of postnatal depression [10, 11].
  Accordingly, we used the General Health Questionnaire-28 (GHQ-28) to assess the level of psychological distress of mothers at month 16 or later after the earthquake. The GHQ-28 is a questionnaire that can provide information on somatic symptoms, anxiety/sleep loss, impaired social activity and depressive tendencies using a single scale. Respondents scoring 6 points or higher on the scale are considered probable cases of psychiatric disorder [12].

## Results

1. Basic attributes of subjects
  The study subjects were women who were either pregnant or were in the early postpartum period (one month or less following delivery) during the earthquake. These women were raising children aged 0–11 months in the 10th month following the earthquake. This would mean that the subject would be a mother of a 4–5 years old child in the 48th month following the earthquake.
  The family size and employment status of the women are provided in Table 1. At 10 months after the earthquake the mean age was 31.8 years (*SD* = 4.9, range = 17–45 years). For 168 subjects (24.8 %) this was their first pregnancy and 496 subjects (73.3 %) had experienced at least one prior pregnancy. The average number of months after having given birth after the earthquake was 5.4 (*SD* = 2.5, range = 0–10 months). Three hundred seventy-five subjects (55.4 %) were employed and 302 subjects (44.6 %) were unemployed.

**Table 1:** Family size and employment status

		Month 10	Month 16	Month 24	Month 36	Month 48
		January 2012	July 2012	March 2013	March 2014	March 2015
n		677	384	351	250	193
Age		31.8 ± 4.9	33.0 ± 4.7	34.0 ± 4.5	34.5 ± 5.0	36.4 ± 4.6
Number of children (%)	1	168 (24.8 %)	―	129 (36.8 %)	―	38 (19.7 %)
	≧2	496 (73.3 %)		222 (63.2 %)		155 (80.3 %)
	Unknown	13 (1.9 %)		0		0
Work (%)	Employed	375 (55.4 %)	―	176 (50.1 %)	128 (51.2 %)	110 (57.0 %)
	Unemployed	302 (44.6 %)		170 (48.4 %)	119 (47.6 %)	83 (43.0 %)
	Unknown	0		5 (1.4 %)	3 (1.2 %)	0
  The mean age at months 16, 24, 36 and 48 after the earthquake were 33.0 (*SD* = 4.7), 34.0 (*SD* = 4.5), 34.5 (*SD* = 5.0) and 36.4 (*SD* = 4.6) years, respectively. At month 24, 129 subjects (36.8 %) had one child and 222 subjects (63.2 %) had two or more children.
  At month 24, 176 subjects (50.1 %) were employed and 170 subjects (48.4 %) were unemployed. At month 36, 128 subjects (51.2 %) were employed and 119 subjects (47.6 %) were unemployed.
2. Mental state of mothers at each postpartum period and related factors
  1. Month 10 after the disaster
    Of the 677 subjects reporting at month 10 after the disaster, 145 postnatal women (21.5 %) had an EPDS score of 9 or higher. In addition, 10 subjects had a score of 20 or higher (Fig. 1).

**Fig. 1:**
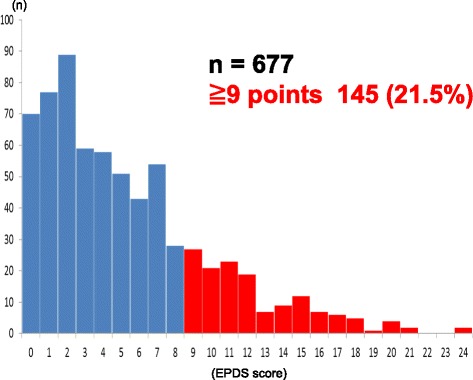
EPDS score 10 months after the disaster. *10–15 % of EPDS takers in JAPAN score over 9 points
    Subjects with high scores were those whose homes were completely destroyed, who were victims of the tsunami, or who had lost employment (either the employment of the subject herself or her husband) (Table 2).

**Table 2:** Association between EPDS score and socioeconomic factors

			EPDS SCORE	*P* value
Work	employed	(375)	4.96 ± 4.38	*P* = 0.012
	unemployed	(302)	5.87 ± 4.89	
Tsunami	experienced	(188)	6.40 ± 5.28	*P* = 0.004
	Not experienced	(487)	4.93 ± 4.30	
Home damage by tsunami	damaged	(79)	6.62 ± 5.53	*P* = 0.01
	undamaged	(595)	5.18 ± 4.48	
Home damage by earthquake	damaged	(17)	8.71 ± 4.93	*P* = 0.024
	undamaged	(657)	5.26 ± 4.60
  2. Month 16 after the disaster
    Of the 384 subjects reporting at month 16 after the disaster, 256 postnatal women (66.7 %) had a GHQ-28 score of 6 or higher (Fig. 2). The related factor found was dissatisfaction with one’s marital life (Table 3).

**Fig. 2:**
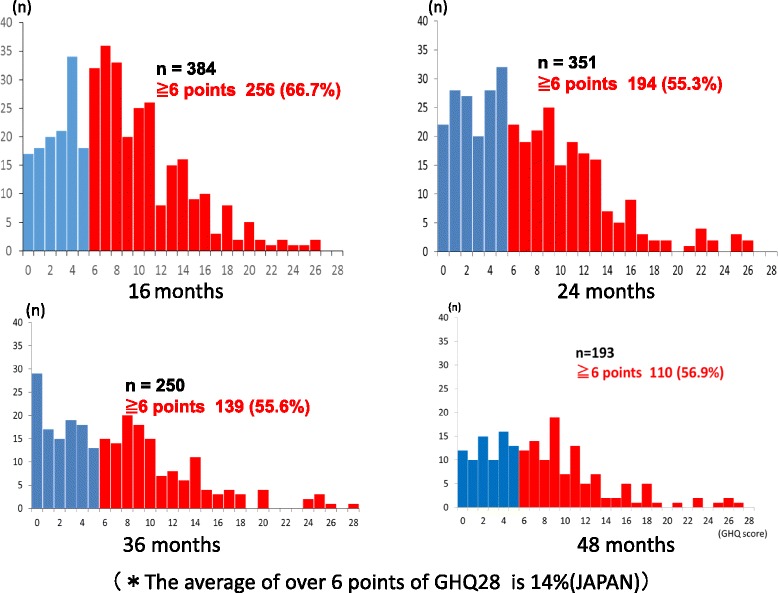
GHQ28 score 16, 24, 36, and 48 months after the disaster. *14 % of GHQ28 takers in Japan score over 6 points

**Table 3:** Association between GHQ-28 score and marital satisfaction

Time after the disaster	Marital satisfaction	GHQ-28 score (*M* ± *SD*)
16 months (*n* = 200)	Dissatisfied	9.5 ± 5.4*
	Satisfied	6.5 ± 4.8
24 months (*n* = 199)	Dissatisfied	11.6 ± 5.4*
	Satisfied	6.0 ± 4.8
36 months (*n* = 232)	Dissatisfied	11.1 ± 7.2**
	Satisfied	6.5 ± 5.1
48 months (*n* = 178)	Dissatisfied	11.1 ± 5.1**
	Satisfied	6.9 ± 5.5

*p < .05. **p < .001
  3. Month 24 after the disaster
    Of the 351 subjects reporting at month 24 after disaster, 194 postnatal women (55.3 %) had a GHQ-28 score of 6 or higher (Fig. 2). The related factor found was dissatisfaction with one’s marital life (Table 4).

**Table 4:** Association between GHQ-28 score, anxiety, and support with childcare

	GHQ-28 score	GHQ-28 score
	36 months	48 months
Economic anxiety	9.1 ± 6.1**	8.6 ± 6.0**
No economic anxiety	4.9 ± 4.3	6.8 ± 5.2
No one to consult about mother’s anxiety	―	16.5 ± 13.4*
Having person to consult about mother’s anxiety		7.6 ± 5.6
No one to support mother with childcare	―	25.0 ± 0.0*
Having person to support mother with childcare		7.6 ± 0.4

*p < .05. **p < .001
  4. Month 36 after the disaster
    Of the 250 subjects reporting at month 36 after the disaster, 139 postnatal women (55.6 %) had a GHQ-28 score of 6 or higher (Fig. 2). Related factors found were financial worries, (economic anxiety) and marital dissatisfaction (Table 4).
  5. Month 48 after the disaster
    Of the 193 subjects reporting at month 48 after disaster, 110 postnatal women (56.9 %) had a GHQ-28 score of 6 or higher (Fig. 2). Related factors found were financial worries, dissatisfaction with one’s marital life and not having support with childcare (Tables 3 & 4).

## Discussion

A characteristic feature of this earthquake is that in addition to the effects of the earthquake itself a giant tsunami struck a wide stretch of the coastal area along the Sanriku coast, sweeping away almost all homes and workplaces. This unprecedented major disaster destroyed the infrastructure supporting residents’ daily lives, and many residents had close friends or family members who were among those killed. It is easy to imagine that these circumstances have left the child-rearing generation with no clear prospects for establishing a future life for themselves, thus increasing their anxiety.

The fact that many of the mothers with a high risk of postnatal depression, according to the EPDS survey at month 10 after the earthquake, were those whose homes had been completely destroyed or had experienced the tsunami tells us about the large scope of the influence of this earthquake. When postnatal depression actually occurs, it is a predisposing factor for attachment disorder [1–6] in the child, and therefore we have been active in providing assistance for persons with high-risk EPDS scores in disaster-affected areas [13–16].

However, continued observation of the mental health of the same set of mothers, using the GHQ-28, showed that the percentage of subjects with high-risk scores remained high over time, with levels of 66.7 % (month 16), 55.3 % (month 24), 55.6 % (month 36), and 56.9 % (month 48). The percentage of Japanese adults with high-risk GHQ-28 scores is 14 % [12], thus the psychological distress among the subjects in the present study is considerably more widespread. (The GHQ-28 data of women raising children is not available) It is likely that the subjects are raising children under such conditions. Examining possible sources of psychological distress as indicated by the high-risk GHQ-28 score, marital dissatisfaction was found at months 16 and 24. In a dangerous situation, it is likely that the degree of trust between a husband and wife will be important. Kubo et al. [17] stated that “in the severely dangerous situation that is a disaster, mothers take the view that they want to rely on their husbands.” Husbands are looked upon to respond to this expectation, but there is little prior research into the mental health or role of fathers during a disaster. It is possible that fathers in such a situation experience psychological distress and marital dissatisfaction as well.

At months 36 and 48, marital dissatisfaction was a factor as well, but in addition there were financial worries. Ten months following the earthquake, the EPDS score of non-working mothers was higher than that of working mothers. The study was conducted at 24, 36, and 48 months following the earthquake and the percentage of working mothers increased at every stage. It is reasonable to assume that mothers had to engage in economic activities. Another aspect of damage to homes from the tsunami and earthquake is the financial burden it has imposed.

Those affected by the disaster have been forced to raise their children in temporary housing, which provides a poor living environment. The financial situation of the child-rearing generation may make it difficult to find a place to live that offers a better environment. In addition, because many places of work were swept away by the tsunami, many people may have lost their place of employment or have jobs that have been suspended for long periods.

The presence of someone to provide support with childcare can help mothers with newborn children maintain a healthy mental state. Scores on the GHQ-28 at month 48 were potentially influenced by having the support of the husband or the mother’s parents. In addition, friends can provide psychological support, even if they do not provide direct or material assistance.

## Conclusion

We conducted a survey, from month 10 after the Great East Japan Earthquake, of women who were pregnant at the time of the earthquake to investigate their mental health during subsequent child-rearing and factors such as postnatal depression and psychological distress. The following results were found.

(1) At month 10 after the earthquake, 21.5 % of the subjects were experiencing postpartum depression. The factors associated with depression were damage suffered from the earthquake (tsunami damage/damage to houses) and loss of employment.

(2) The percentage of subjects that were experiencing psychological distress at months 16, 24, 36, and 48 after the earthquake were 66.7, 55.3, 55.6 and 56.9 %, respectively, and the factors associated with their distress were marital dissatisfaction, financial worries, and not having childcare support.

Based on this, even though four years have passed since its occurrence, The Great East Japan Earthquake is anticipated to continue to have a huge effect on the psychophysical condition of mothers raising children. This will necessitate continued financial aid along with childcare and educational support, such as ‘child rearing techniques and communication within the marriage’.
